# Utility and Outcomes of a Keystone Perforator Island Flap for Reconstruction at Various Anatomical Locations: A Prospective Study

**DOI:** 10.7759/cureus.71644

**Published:** 2024-10-16

**Authors:** Yahia A Alsiaghi, Mohaned Y Al-ajaly, Majed Y Al-Warafi, Haitham M Jowah

**Affiliations:** 1 Department of Plastic Surgery, Typical Model Hospital, Sana'a City, YEM; 2 Department of Plastic Surgery, Al-Khamsin Hospital, Sana'a City, YEM; 3 Department of Plastic Surgery, Al-Moutawakel Hospital, Sana'a City, YEM; 4 Department of Surgery, 21 September University, Sana'a City, YEM; 5 Department of Surgery, Sana'a University, Sana'a, YEM

**Keywords:** keystone island perforator flap, plastic surgery, resource-constrained settings, soft tissue reconstruction, trauma, yemen

## Abstract

Background

In Yemen, traumatic wounds are prevalent, imposing a substantial burden on plastic surgery teams operating within limited-resource settings. Advanced microsurgical reconstruction options are scarce, and expertise in free flap techniques is limited. While local flaps are commonly used for soft tissue reconstruction, there is a need for simpler, effective alternatives with lower complication rates. This study aimed to evaluate the effectiveness and outcomes of the keystone island perforator flap (KIPF) as a viable alternative for soft tissue coverage in resource-challenged facilities.

Methods

From February 2021 to December 2023, a single surgeon's team in three plastic and reconstructive surgery departments in Sana'a, Yemen, conducted this prospective study, which included 35 patients who underwent KIPF for various soft tissue defects. We assessed patient demographics, intraoperative variables, and postoperative outcomes, including aesthetic outcomes evaluated using the Patient and Observer Scar Assessment Scale (POSAS) at six months postoperatively. We analyzed the factors influencing flap success and complication rates.

Results

The mean age of patients was 29 ± 11.75 years, with the majority being males, comprising 86% of the sample. Trauma was the leading cause of tissue defects (77.1%), primarily affecting the legs (57.14%) and feet (11.43%). The mean operative time was 73.57 minutes. Complications occurred in 26% of patients, with flap dehiscence being the most common (11.43%). Ninety-seven percent of patients experienced flap survival, and 91.4% experienced complete wound healing within a median of 16 days. Scar assessments at six months postoperatively indicated satisfactory aesthetic outcomes, with scars resembling normal skin.

Conclusion

The KIPF technique proves to be a reliable and practical option for soft tissue reconstruction in resource-limited settings. Its simplicity, minimal postoperative care needs, and high flap survival rates make it an advantageous alternative to more complex reconstructive techniques. This study supports the adoption of KIPF as a standard approach in similar environments with limited reconstructive surgery capabilities.

## Introduction

Soft tissue reconstruction involves various reconstructive options influenced by factors such as defect size, location, condition of underlying structures, and the availability of local soft tissue [1,2]. In Yemen, a country with constrained resources, the high incidence of traumatic wounds from injuries and accidents places a substantial burden on plastic surgery teams. The lack of advanced microsurgical centers and limited expertise in free flap techniques necessitate the use of local flaps for reconstruction [3].

While local flaps are essential tools in such settings, they can present challenges, particularly for less experienced surgeons, as they often require a steep learning curve and may be associated with high complication rates [4]. To address these limitations, the keystone island perforator flap (KIPF) technique has gained recognition as a valuable method in resource-limited settings like Yemen. This technique offers a reliable, efficient solution for fasciocutaneous closure with minimal postsurgical care requirements, making it a practical alternative to more complex procedures [5,6]. The KIPF is advantageous for its ease of dissection, preservation of perforating vessels, and low donor site morbidity, with studies reporting high success rates and patient satisfaction [7,8]. However, like any reconstructive option, the KIPF is not without risks; complications such as flap necrosis and wound infection may occur, depending on factors like perforator anatomy, flap design, and surgical technique [9, 10]. Its versatility has made it suitable for reconstructing defects in various anatomical regions, particularly on the trunk and lower extremities [9,11-13].

Given the limited research on KIPF in Yemen, this study aimed to evaluate the effectiveness, success rate, aesthetic outcomes, and complications associated with KIPF across different anatomical locations. By investigating its utility and safety in a resource-constrained environment, this study aims to provide insights to guide surgical decision-making and enhance patient care in similar settings.

This article was posted on the Research Square preprint server on August 27, 2024.

## Materials and methods

This prospective case series study was conducted from February 2021 to December 2023 at three plastic and reconstructive surgery departments: Police Typical Hospital, Al-Moutawakel Hospital, and Al-Khamsin Hospital in Sana'a, Yemen.

The study included patients with soft tissue defects in various anatomical locations who underwent reconstruction using the KIPF by a single experienced plastic surgeon's team. Patients were eligible if they had soft tissue defects suitable for KIPF, provided informed consent, and were capable of participating in follow-up visits. The exclusion criteria were patients who did not undergo reconstruction via KPIF, those who did not provide informed consent, and those with soft tissue defects that were not appropriate for KPIF due to size, location, or comorbid conditions.

Patient demographic data, including age, sex, and underlying etiologies of soft tissue defects, were collected. Detailed information on defect characteristics, such as size, location, and associated injuries, was documented. Surgical details, including the type of flap, dimensions, and intraoperative findings (such as blood loss and operative time), were meticulously recorded. Postoperative outcomes such as complications, flap survival, wound healing time, and length of hospital stay were monitored. Data collection was standardized using structured forms to ensure consistency.

The primary outcome was the success rate of the KIPF procedure, which was defined as complete flap survival without necrosis or significant complications. Aesthetic outcomes were evaluated using the Patient and Observer Scar Assessment Scale (POSAS 3.0), which includes both patient-reported and observer-reported assessments of scar appearance [14]. The secondary outcomes included complication rates, which were categorized as minor (e.g., partial flap necrosis, seroma formation) or major (e.g., complete flap failure, significant infection).

The surgical procedure begins with careful preoperative planning [7]. We mark the surgical site to align the flap along relaxed skin tension lines, thereby minimizing closure tension and optimizing healing. We accurately measure the defect and design the flap adjacent to it as a trapezoidal or elliptical shape, with dimensions typically 1.5 to 2 times the defect width to ensure adequate coverage. We align the flap's long axis parallel to the defect and orient its short axis to maximize tissue laxity.

Once we finalize the flap design, we make incisions along the marked lines. To preserve perforator vessels and ensure a robust blood supply to the flap, we elevate it in a subfascial or subcutaneous plane. This vascular preservation is a key advantage of the KIPF technique, reducing the need for extensive dissection and enhancing flap viability.

Following elevation, we carefully mobilize and advance the flap to cover the defect. We then inset the flap into the defect and secure it with interrupted or continuous sutures, ensuring even tension distribution along the suture line to maintain blood flow. We carefully manage tension at all points to prevent ischemia and selectively perform any additional undermining to facilitate optimal flap positioning.

Depending on the size and location of the defect, either primary closure of the donor site or skin grafting may be necessary. We apply a sterile dressing to protect the wound and promote healing. We may utilize negative pressure wound therapy in some cases, especially for larger defects or areas with significant tissue loss, to promote faster healing and reduce complications.

**Figure 1:**
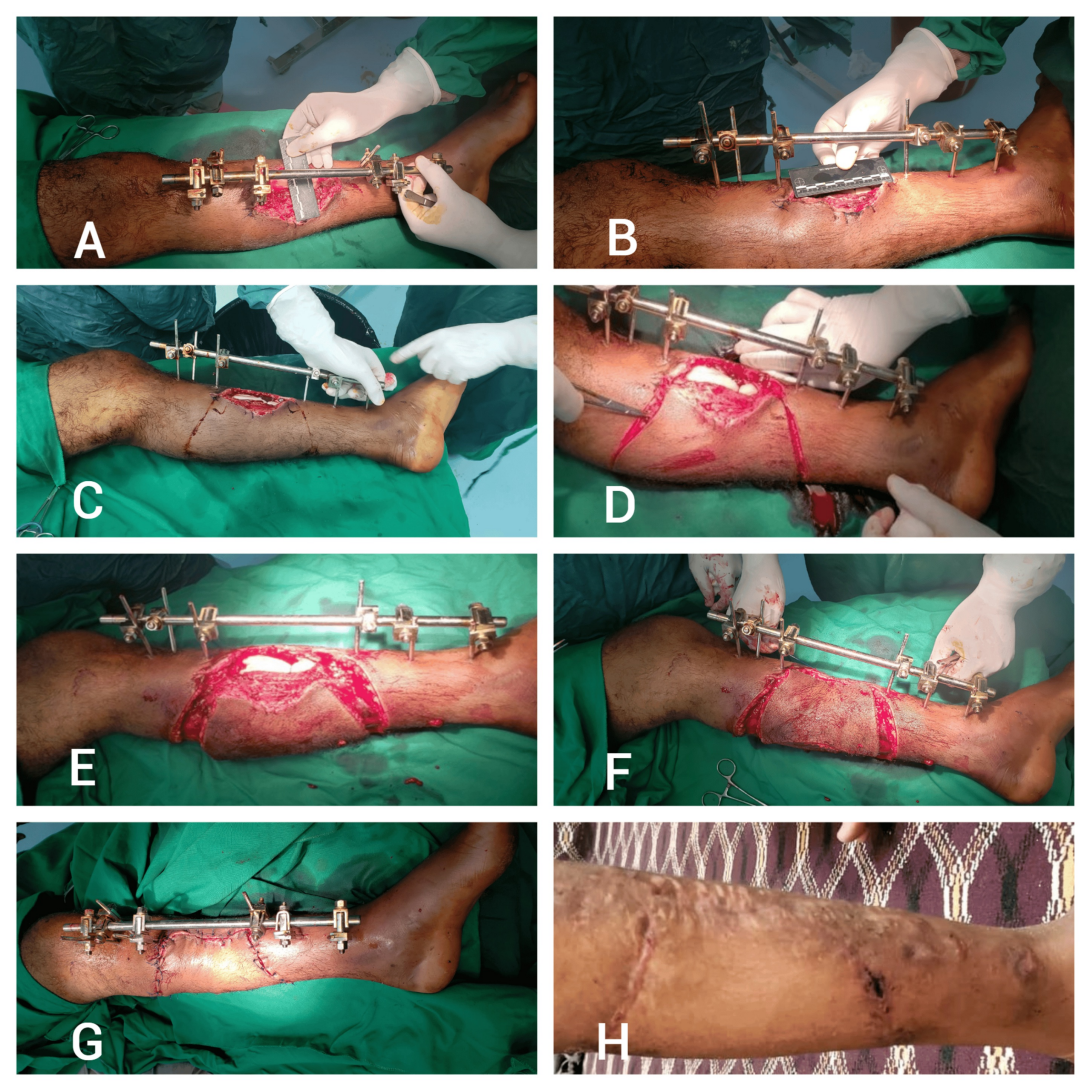
Patient one: a 35-year-old male with a post-gunshot injury to the left proximal tibia Sequential steps of the keystone perforator island flap (KPIF) for soft tissue reconstruction, specifically demonstrating type II A, B) Measurement of defect size; C) Flap design with elliptical marking adjacent to the defect; D) Incision along the margins for flap elevation E); Flap elevation in the subcutaneous plane, preserving perforator vessels; F) Flap advancement and in setting over the defect with sutures; G): Complete closure of the defect and donor site, with a sterile dressing applied H) Six-month follow-up showing a well-integrated, healed flap

In postoperative care, we monitor the flap for signs of ischemia or necrosis and educate patients on wound care procedures. We initially follow up with patients every three days for the first two weeks and then once a month for the next six months to assess long-term outcomes. This protocol outlines the general steps for all KPIF types, with Figure 1 showcasing a type II flap as a representative example.

Patients followed a comprehensive follow-up schedule that monitored healing and promptly identified any complications. In the first two weeks post-surgery, patients attended visits every three days for wound assessment, dressing changes, and suture removal. After this period, we scheduled monthly visits for six months to assess long-term outcomes such as flap survival, wound healing, and scar maturation.

We used the Patient and Observer Scar Assessment Scale (POSAS) to assess the aesthetic outcomes of scars at the six-month follow-up visit. Patients completed the Patient Scar Assessment Scale (PSAS), which provides a subjective evaluation of scar appearance, covering aspects such as color, pliability, and thickness. An experienced plastic surgeon, independent of the surgical team, completed the Observer Scar Assessment Scale (OSAS) to offer an objective evaluation of the scar [14]. This dual approach ensured a thorough assessment of scar aesthetics from both the patient's perspective and an expert's clinical viewpoint.

We promptly implemented interventions in cases of complications, such as partial flap necrosis, seroma, or infection. We managed partial flap necrosis with bedside debridement and local wound care, and under sterile conditions, we aspirated seromas. We treated any infections with antibiotics and addressed major complications, such as complete flap failure, with revision surgery when necessary.

We analyzed the data using SPSS version 26 (IMB Inc., Armonk, New York). Descriptive statistics summarized patient demographics, intraoperative details, and postoperative outcomes. We applied Chi-squared tests and Fisher's exact tests to compare success rates across different groups (e.g., defect location, flap size). We used univariate analysis to identify factors associated with flap success, including defect size, location, and comorbidities. Complication rates were calculated and compared using appropriate statistical methods.

We analyzed the aesthetic assessment results by calculating the mean POSAS scores. We evaluated the interrater reliability of the POSAS ratings using intraclass correlation coefficients (ICCs). A p-value of less than 0.05 was considered statistically significant.

The study received ethical approval from the Institutional Review Board of the Yemeni Council of Health Specialization (Approval Number: IRB-2024-1234). We obtained informed written consent from all participants in compliance with the Declaration of Helsinki. We informed participants of their right to withdraw from the study at any time, and we treated all data with strict confidentiality.

## Results

The study included 35 patients who underwent KIPF for reconstruction at various anatomic locations. The mean age was 29 ± 11.75 years, ranging from 10-60 years, with 22.9% of patients aged ≤18 years, 74.3% aged 19-50 years, and 2.9% aged >50 years. Sex distribution was male (85.7%), with 14.3% of patients being female. The special habits of the participants included qat chewing (74.3%), smoking (34.3%), and Shama use (25.7%). Most patients (80%) had no comorbidities, whereas 5.7% had diabetes mellitus (DM) and lower limb paralysis, with other conditions observed in 2.9% of patients. Trauma or accidents accounted for 77.1% of tissue defects, followed by pressure sores and burns/scarring (5.7% each; Table 1).

**Table 1:** Demographic and clinical characteristics of the study population (N=35)

Characteristic	Category	Frequency	Percentage
Age group	≤18 years	8	22.9
	19-50 years	26	74.3
	>50 years	1	2.9
Mean ± SD		29 ± 11.75	
Sex	Male	30	85.7
	Female	5	14.3
Special habits	Qat chewing	26	74.3
	Smoking	12	34.3
	Shama using	9	25.7
Comorbidities	No comorbidity	28	80
	Diabetes mellitus	2	5.7
	Lower limb paralysis	2	5.7
	Hypertension (HTN)	1	2.9
	Deep vein thrombosis (DVT)	1	2.9
	Cerebrovascular accident (CVA)	1	2.9
	Psychological issues	1	2.9
Etiology of defects	Trauma/accident	27	77.1
	Pressure sore	2	5.7
	Burn/scarring	2	5.7
	Infection/osteomyelitis	1	2.9
	Oncologic cause	1	2.9
	Ulcer	1	2.9
	Myelomeningocele	1	2.9

The preoperative assessment indicated that the leg was the most common site of defect, accounting for 57.14% of the defects, followed by the foot (11.43%) and the thigh and back (8.57% each). Other locations were less common. Primary closure was attempted in 25.71% of the patients. The mean size of the first defects was 4.44 cm in width and 8.74 cm in length, whereas the second defects had a mean width of 4 cm and a mean length of 7.67 cm (Table 2).

**Table 2:** Anatomical locations and dimensions of tissue defects (N=35)

Characteristic	Category	Frequency	Percentage
Defect location	Leg	20	57.14
	Foot	4	11.43
	Thigh	3	8.57
	Back	3	8.57
	Buttock	2	5.71
	Upper limbs	2	5.71
	Abdomen	1	2.86
Primary closure attempts	Yes	9	25.71
Defect Sizes	1st defect width (mean ± SD)	4.44 ± 1.46	
	1st defect length (mean ± SD)	8.74 ± 2.51	
	2nd defect width (mean ± SD)	4 ± 2	
	2nd defect length (mean ± SD)	7.67 ± 1.15	
	Median number of defects	1 (1-2)	

The mean operative time was 73.57 ± 17.56 minutes. Behan's classification classified the majority of flaps as type IIA (54.29%), a double keystone flap with the advancement of both edges, followed by type I (22.86%) and type III (14.29%). Types IIB and IV were less common, representing 5.71% and 2.86% of cases, respectively.

The average dimensions of the first flap were 6.03 cm in width and 10.94 cm in length, while the second flap averaged 4.63 cm in width and 8.29 cm in length. In all of the cases, a red dotted sign was seen during the surgery, which means that the flaps' blood flow was stable. This showed that the flaps had consistent vascularity throughout the procedures (Table 3).

**Table 3:** Intraoperative assessments and flap characteristics (N=35) This table includes the classification of flaps based on Behan's classification system, which categorizes keystone perforator island flaps (KIPF) into several types: type I refers to a single keystone flap; type IIA involves a double keystone flap with the advancement of both edges; type IIB describes a double keystone flap with a rotation advancement; type III is characterized by a keystone flap with V-Y advancement; and type IV involves bilobed or multiple keystone flaps used for large defects

Intraoperative variable	Category	Frequency	Percentage
Operative time (mean ± SD)		73.57 ± 17.56	
Behan's classifications	Type IIA	19	54.29
	Type I	8	22.86
	Type III	5	14.29
	Type IIB	2	5.71
	Type IV	1	2.86
Total number of flaps		1 (1-2)	
Flap sizes	1st flap width (mean ± SD)	6.03 ± 1.77	
	1st flap length (mean ± SD)	10.94 ± 3.05	
	2nd flap width (mean ± SD)	4.63 ± 2.50	
	2nd flap length (mean ± SD)	8.29 ± 2.06	
Flap hemodynamic changes	Red dotted sign	35	100

Postoperative complications occurred in 26% of patients, with flap dehiscence being the most common (11.43%). Other complications included epidermolysis, marginal flap necrosis, venous congestion, seroma, and flap infection (each 2.86%). Reoperation was necessary for 5.7% of patients. The management strategies included bedside closure of dehiscent flaps (8.56%), skin grafting (2.86%), and other interventions. The median length of stay was three days, with most flaps fully surviving (97%) and complete wound healing occurring in 91.4% of the patients within a median time of 16 days (Table 4).

**Table 4:** Postoperative outcomes and complications (N=35)

Postoperative Variable	Category	Frequency	Percentage
Postop complications	Flap dehiscence	4	11.43
	Epidermolysis/ skin necrosis	1	2.86
	Marginal flap necrosis	1	2.86
	Venous congestion	1	2.86
	Seroma	1	2.86
	Flap infection	1	2.86
	Reoperation	2	5.7
Complication management	Bedside closure of a dehiscent flap	3	8.56
	Full-thickness skin graft	1	2.86
	Bedside seroma evacuation	1	2.86
	Debridement and readvancement	1	2.86
	Conservative management	3	8.56
Postoperative outcomes	LOS (median, IQR)	3 (2-7) days	
	Flap survival	Fully survived	34
		Partially survived	1
	Time to complete healing (median, IQR)	16 (12-30) days	

Esthetic outcomes according to the POSAS scale

Patient Scar Assessment Sscale (PSAS)

The reliability of the Patient Scar Assessment Scale (PSAS) was confirmed, with a high Cronbach's alpha of 0.907 for 17 items, whereas the observer-reported outcome scale also demonstrated excellent reliability, with a Cronbach's alpha of 0.913 across 10 items. Descriptive analysis revealed that patients reported minimal esthetic concerns and physical discomfort related to the scars. The scars were assessed as slightly different from normal skin in terms of color, shininess, elevation, hardness, irregularity, and edge appearance (Table 5).

**Table 5:** Patient scar assessment at the current moment according to the Patient Scar Assessment Scale (PSAS) This table presents the results of the Patient Scar Assessment Scale (PSAS) at the current moment, evaluating various aspects of scar appearance and texture as reported by the patients, compared to their normal skin. The table includes the frequency and percentage of patient responses across different severity levels (extremely, severely, moderately, minimally, not) for characteristics such as color difference, shininess, raised/sunken scars, hardness, irregularity, and whether the scar appears stretched or widened. The mean scores and standard deviations (SD) for each characteristic are also provided, reflecting the overall esthetic and physical impact of the scars on the patients. This assessment is crucial for understanding patient perceptions of scar outcomes in relation to their normal skin following the reconstructive procedures.

Parameter	Extremely	Severely	Moderately	Minimally	Not	Mean ± SD
Color difference	0 (0%)	1 (3%)	13 (37%)	21 (60%)	0 (0%)	2.43 ± 0.56
Shininess	0 (0%)	1 (3%)	3 (9%)	16 (46%)	15 (43%)	1.71 ± 0.75
Raised/sunken scar	0 (0%)	0 (0%)	5 (14%)	25 (71%)	5 (14%)	2.00 ± 0.54
Hardness	0 (0%)	1 (3%)	12 (34%)	15 (43%)	7 (20%)	2.20 ± 0.80
Irregularity	0 (0%)	0 (0%)	11 (31%)	22 (63%)	2 (6%)	2.26 ± 0.56
Stretched or widened	0 (0%)	0 (0%)	7 (20%)	13 (37%)	15 (43%)	1.77 ± 0.77

In addition, patients experienced minimal physical discomfort, including rare cases of sensitivity, numbness, and pain. Overall, a mean score of 1.69 (SD=0.58) reflects positive esthetic and physical outcomes for the scars (Table 6).

**Table 6:** Patient scar assessment during the last week according to the Patient Scar Assessment Scale (PSAS) Data are presented as frequency and percentage

Parameter	Extremely	Severely	Moderately	Minimally	Not	Mean ± SD
Sensitivity	0 (0%)	0 (0%)	3 (9%)	8 (23%)	24 (69%)	1.40 ± 0.65
Numbness	0 (0%)	0 (0%)	1 (3%)	16 (46%)	18 (51%)	1.51 ± 0.56
Pain	0 (0%)	0 (0%)	0 (0%)	4 (11%)	31 (89%)	1.11 ± 0.32
Shooting sensation	0 (0%)	0 (0%)	1 (3%)	5 (15%)	28 (82%)	1.21 ± 0.48
Burning sensation	0 (0%)	0 (0%)	0 (0%)	21 (60%)	14 (40%)	1.60 ± 0.50
Itch	0 (0%)	0 (0%)	4 (11%)	23 (66%)	8 (23%)	1.89 ± 0.58
Tingling	0 (0%)	0 (0%)	0 (0%)	5 (16%)	27 (84%)	1.16 ± 0.37
Scar tightness at rest	0 (0%)	0 (0%)	2 (6%)	13 (37%)	20 (57%)	1.49 ± 0.61
Tightness with movement	0 (0%)	0 (0%)	9 (26%)	17 (49%)	9 (26%)	2.00 ± 0.73
Fragility	0 (0%)	0 (0%)	1 (3%)	4 (11%)	30 (86%)	1.17 ± 0.45
Dryness	0 (0%)	0 (0%)	5 (14%)	16 (46%)	14 (40%)	1.74 ± 0.70

Observer Scar Assessment (OSAS)

The observer-reported outcome scale exhibited excellent internal consistency, with a Cronbach's alpha of 0.913 across the 10 items, confirming its reliability. Descriptive analysis of the observer scale revealed that scars were characterized by hypopigmentation (68.6%), pale/white vascularity (74.3%), depressed surface level (80%), and irregular texture (88.6%; Table 7).

**Table 7:** Descriptive analysis of scar characteristics according to the Observer Scar Assessment Scale (OSAS) This table provides a descriptive analysis of scar characteristics as evaluated by observers using the Observer Scar Assessment Scale (OSAS). The table includes the frequency and percentage of different scar features, such as pigmentation (e.g., hypopigmentation, hyperpigmentation, mixed pigmentation), vascularity (e.g., pale/white, pink, red, purple), surface level (elevated or depressed), and texture (irregular or overly smooth). These characteristics are essential for objectively assessing the quality of scar healing and comparing the observed scar attributes with normal skin. The OSAS offers a standardized method for evaluating the cosmetic and structural aspects of scars, which is vital for determining the success of reconstructive procedures.

Variable	Category	Frequency	Percentage (%)
Pigmentation	Hypopigmentation	24	68.6
	Hyperpigmentation	2	5.7
	Mixed	9	25.7
Vascularity	Pale/white	26	74.3
	Pink	8	22.9
	Red	2	5.7
	Purple	1	2.9
Surface level	Elevated	7	20
	Depressed	28	80
Texture	Irregular	31	88.6
	Overly smooth	4	11.4

Most observers rated the overall quality of scars as minimally different from that of normal skin, with an overall mean score of 2.15 (SD=0.65) across various characteristics, suggesting favorable healing progress and acceptable cosmetic outcomes (Table 8).

**Table 8:** Observer's scar assessment according to the Observer Scar Assessment Scale (OSAS) Data are presented as frequency and percentage.

Parameter	Extremely	Severely	Moderately	Minimally	Not	Mean ± SD
Overall quality	0 (0%)	1 (3%)	14 (40%)	20 (57%)	0 (0%)	2.46 ± 0.56
Pigmentation	0 (0%)	1 (3%)	10 (29%)	24 (69%)	0 (0%)	2.34 ± 0.54
Vascularity	0 (0%)	1 (3%)	4 (11%)	28 (80%)	2 (6%)	2.11 ± 0.53
Surface level	0 (0%)	0 (0%)	9 (26%)	20 (57%)	6 (17%)	2.09 ± 0.66
Scar texture	0 (0%)	2 (6%)	11 (31%)	21 (60%)	1 (3%)	2.40 ± 0.65
Firmness	0 (0%)	1 (3%)	11 (31%)	22 (63%)	1 (3%)	2.34 ± 0.59
Adherence	0 (0%)	0 (0%)	6 (17%)	15 (43%)	14 (40%)	1.77 ± 0.73
Scar tension	0 (0%)	0 (0%)	11 (31%)	16 (46%)	8 (23%)	2.09 ± 0.74
Edges widened	0 (0%)	0 (0%)	9 (26%)	13 (37%)	13 (37%)	1.89 ± 0.80
Scar marks	0 (0%)	1 (3%)	5 (14%)	20 (57%)	9 (26%)	1.94 ± 0.73

Correlation Between OSAS and PSAS

Spearman's correlation analysis revealed significant correlations between patient-reported outcomes and observer assessments, indicating strong agreement between patients' perceptions and observers' evaluations, particularly in areas such as color differences, firmness, and scar tension (Table 9).

**Table 9:** Selected spearman correlation coefficients between patient-reported and observer-reported outcomes ** indicates significance at the 0.01 level (2-tailed), and * indicates significance at the 0.05 level (2-tailed)

Patient-reported outcome	Observer-reported outcome	Correlation coefficient	p-value
Color difference	Overall Quality of the Scar	0.600**	0.000**
Shininess	Firmness	0.616**	0.000**
Surface irregularity	Scar Texture	0.425*	0.011*
Edges widened	Adherence	0.437**	0.008**
Tension at rest	Scar Tension	0.780**	0.000*

Selected case presentations and reconstructive outcomes

Patient Two

**Figure 2:**
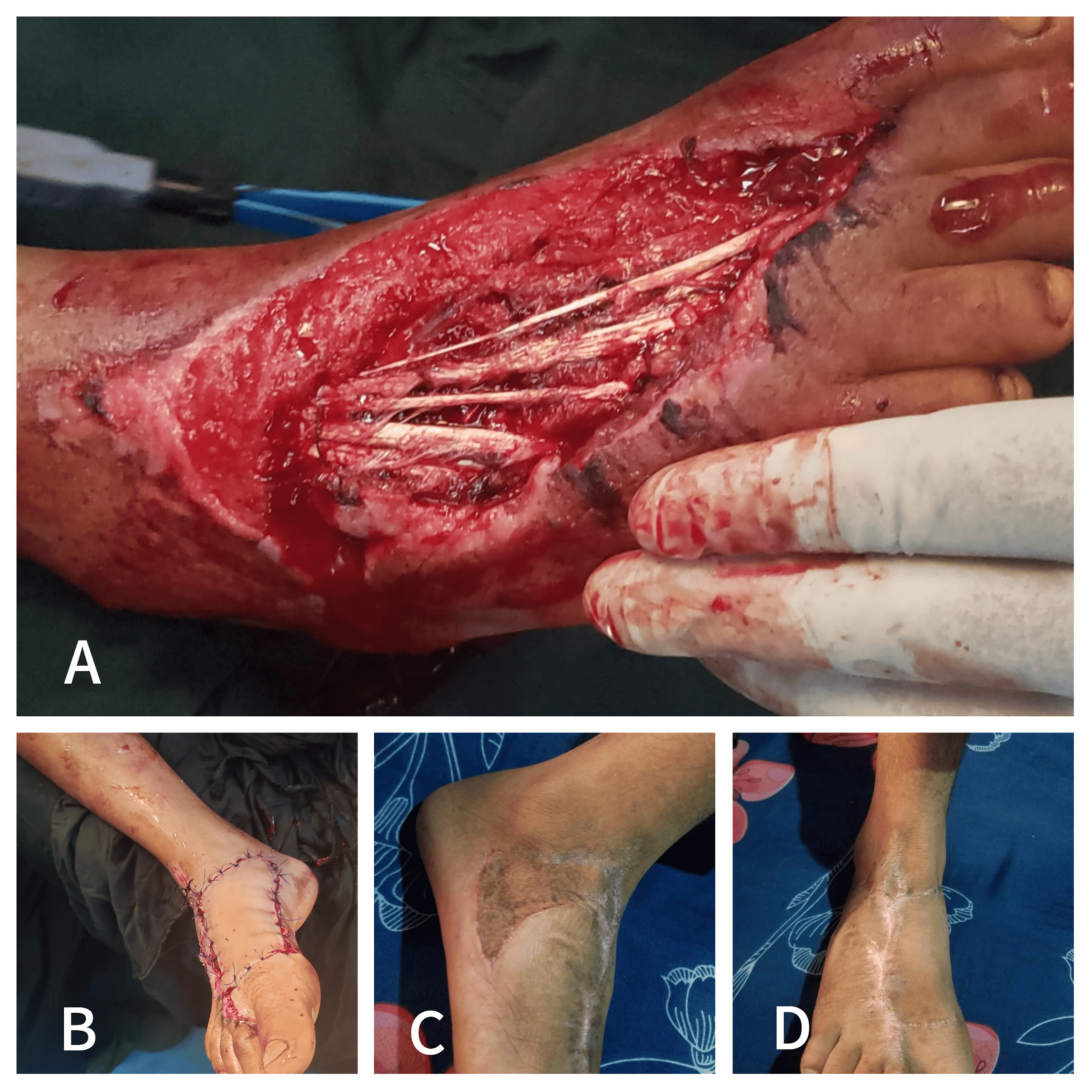
Patient two - a 13-year-old male patient presented following a road traffic accident with a crush injury on the dorsum of the right foot with exposed tendons A) Preoperative image showing a crush injury on the dorsum of the right foot, revealing exposed tendons; B) Immediate postoperative view following the type III keystone island perforator flap (KIPF) procedure, demonstrating the closure of the defect; C) Six-month postoperative image showing the dorsal aspect of the foot with a well-healed scar, exhibiting slight hypopigmentation; D) Lateral view at six months postoperatively, highlighting the scar's minimal difference from the surrounding normal skin, indicating favorable healing and satisfactory cosmetic outcomes

A 13-year-old male patient presented following a road traffic accident with a crush injury on the dorsum of the right foot, measuring approximately 6 × 4 cm. The injury resulted in exposed tendons, necessitating surgical intervention. The patient underwent a type III keystone island perforator flap (KIPF) procedure to achieve coverage and promote healing. At the six-month postoperative follow-up, the scar was observed to be hypopigmented with minimal difference compared to the surrounding unaffected skin. The scar was assessed using the Observer Scar Assessment Scale (OSAS), showing favorable cosmetic outcomes. The patient also reported high satisfaction with the scar's appearance according to the Patient Scar Assessment Scale (PSAS), indicating a successful reconstructive outcome (Figure 2).

Patient Three

**Figure 3:**
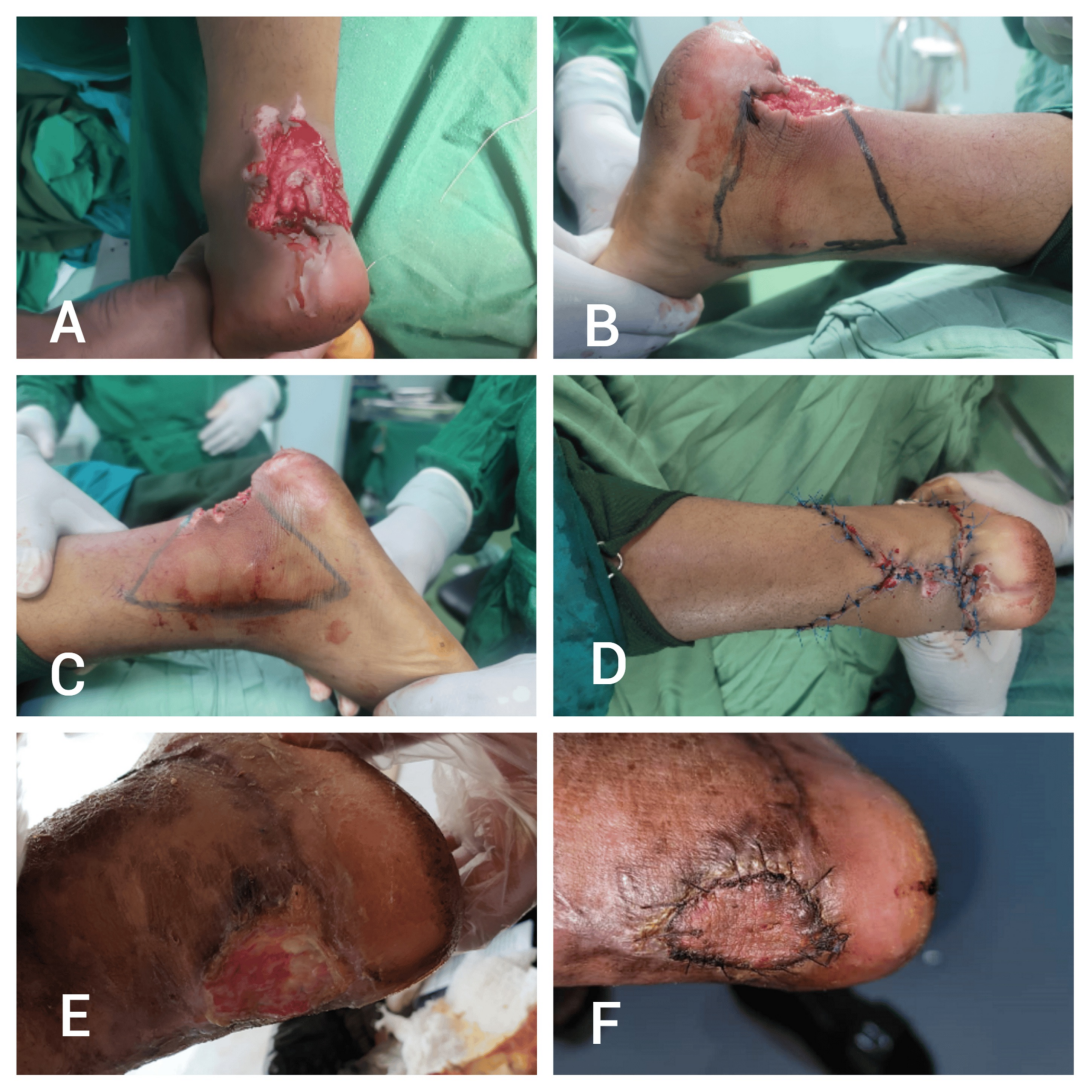
Patient three - an 18-year-old patient presented with a left Achilles tendon injury following a road traffic accident (RTA) A) Preoperative image showing the left Achilles tendon injury with a soft tissue defect measuring 7 × 4 cm and separation from the insertion site; B) Intraoperative marking for the planned type III keystone island perforator flap (KIPF) procedure; C) Elevation of the KIPF, illustrating the flap design and preparation for defect coverage; D) Immediate postoperative view demonstrating the closure of the defect following the KIPF procedure; E) Postoperative complication showing wound dehiscence with marginal necrosis of the flap; F) Follow-up image after treatment with daily dressings and a full-thickness skin graft, showing the final appearance of the scar.

An 18-year-old patient presented with a left Achilles tendon injury following a road traffic accident (RTA). The injury included separation from the tendon’s insertion site and a soft tissue defect measuring 7 × 4 cm. The patient underwent a type III keystone island perforator flap (KIPF) procedure for tendon repair and soft tissue coverage. Postoperatively, the patient experienced complications, including wound dehiscence and marginal necrosis of the flap. These complications were managed with daily dressings and eventually a full thickness skin graft. At the six-month follow-up, the patient reported a hyperpigmented, purple, elevated scar and expressed overall dissatisfaction with the cosmetic outcome. The observer also noted moderate differences between the scar and the surrounding unaffected skin (Figure 3).

Patient Four

**Figure 4:**
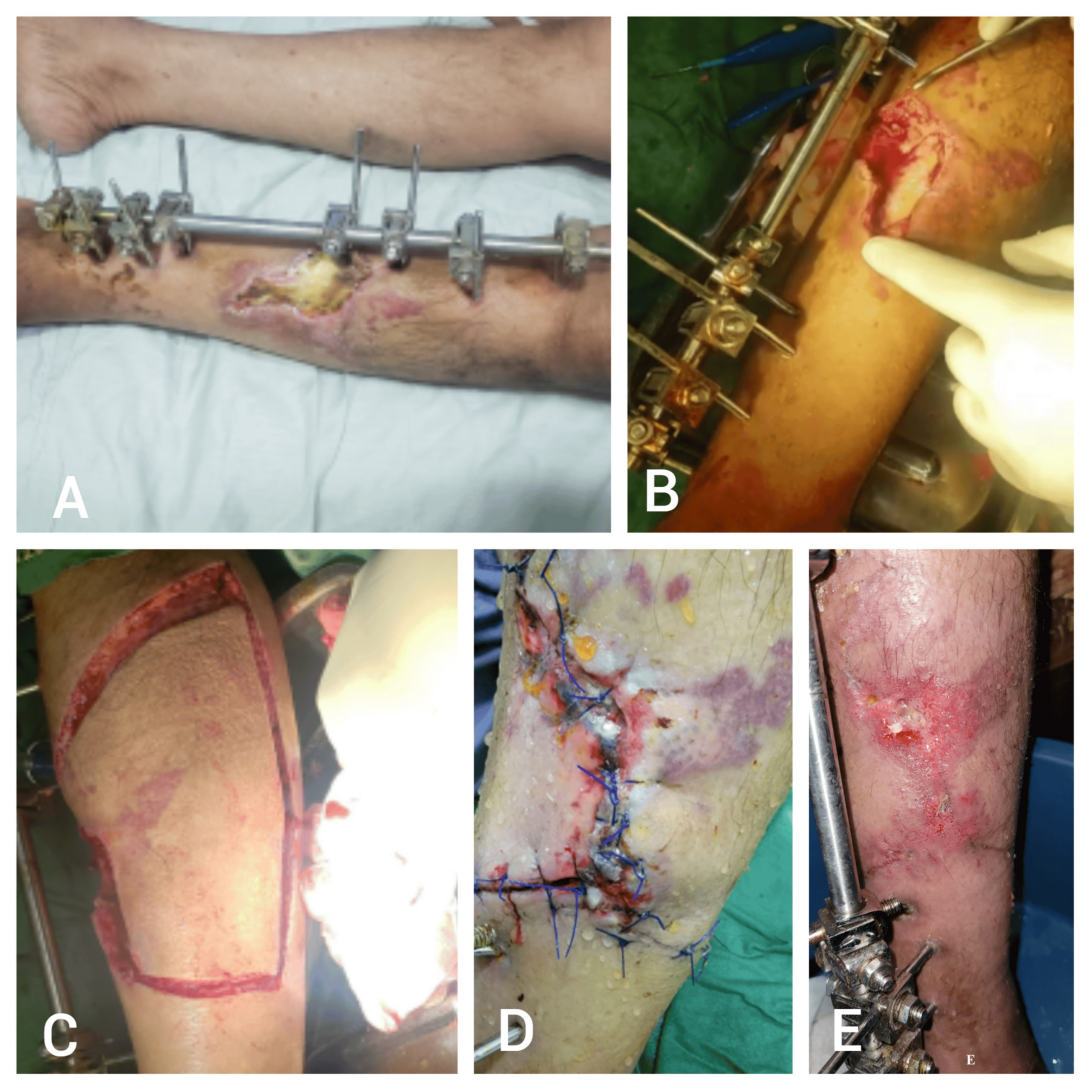
Patient four - a 60-year-old male patient with a history of hypertension (HTN) and diabetes mellitus (DM) presented following a road traffic accident (RTA) with trauma to the left leg A) Preoperative image showing the left leg with a compound tibial and fibular fracture, along with a soft tissue defect measuring 10 × 4 cm, and external fixation in place; B) Intraoperative debridement of the necrotic tissue; C) Elevation and preparation of the type IIA keystone island perforator flap (KIPF) for coverage of the defect. (D) Postoperative complication of epidermolysis and skin necrosis of the flap. (E) Image showing the wound after management with daily dressings, revealing the final appearance at follow-up.

A 60-year-old male patient with a history of hypertension (HTN) and diabetes mellitus (DM) presented following a road traffic accident (RTA) with trauma to the left leg. The injury resulted in a compound fracture of the tibia and fibula, accompanied by soft tissue and skin necrosis, leading to a soft tissue defect measuring 10 × 4 cm. The patient underwent surgical debridement of the necrotic tissue followed by reconstruction using a type IIA keystone island perforator flap (KIPF). Postoperatively, the patient developed complications, including epidermolysis and partial necrosis of the flap, which were managed with daily dressings. At the 6-month follow-up, the patient reported mild dissatisfaction with the cosmetic outcome, and the observer noted a moderate difference between the scar and the surrounding unaffected skin (Figure 4).

Patient Five

**Figure 5:**
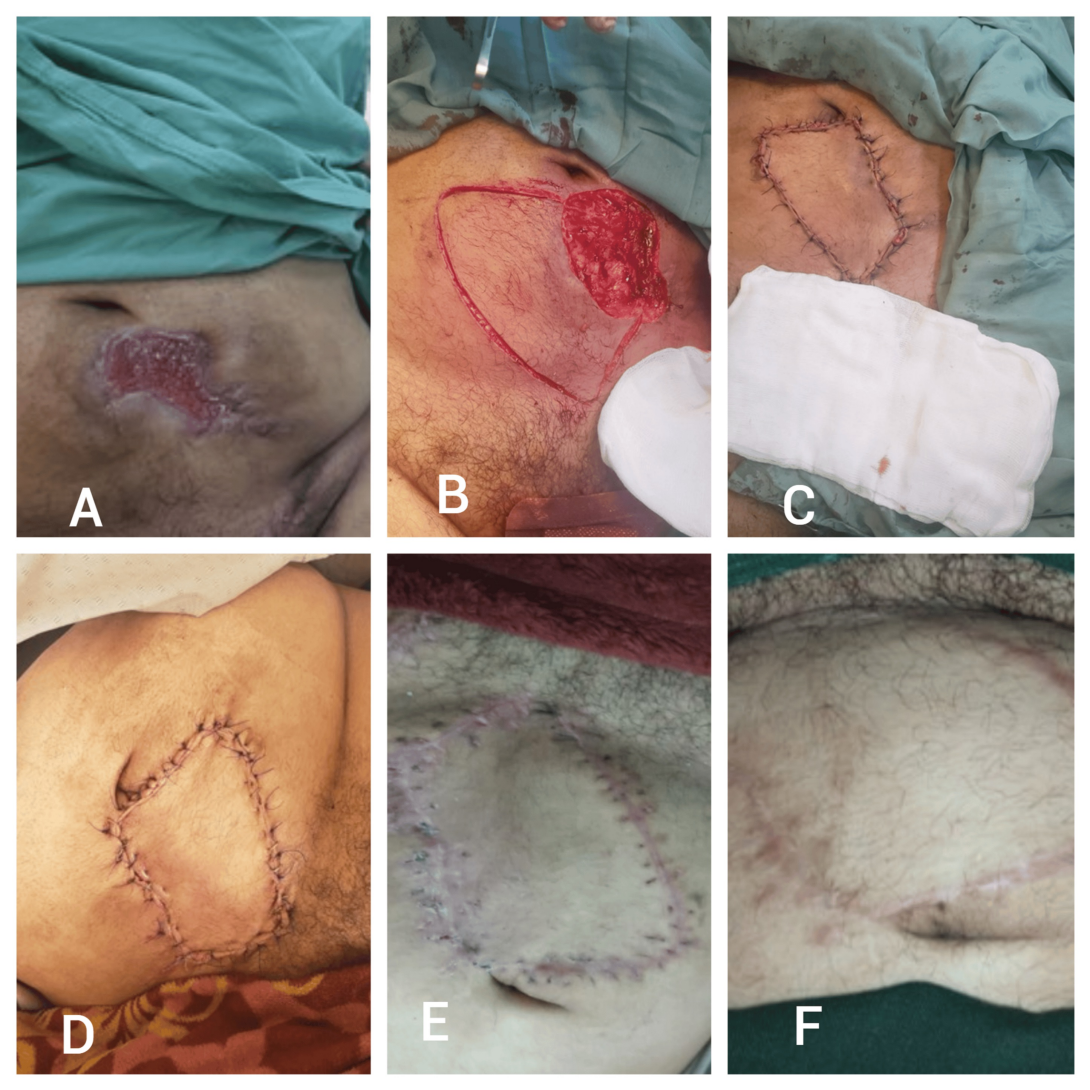
Patient five - a 40-year-old man presented with lower abdominal wall wound following a gunshot wound injury A) Preoperative image showing the lower abdominal wall wound; B) Intraoperative view after debridement and planning of the type I keystone island perforator flap (KIPF) for reconstruction; C) Immediate postoperative view after flap closure, showing the initial alignment of the wound edges; D) Early postoperative image demonstrating satisfactory wound healing; E) Follow-up image showing further maturation of the scar; F) Final follow-up at several months, revealing a well-healed scar with minimal difference from the surrounding skin.

A 40-year-old man with a history of smoking and qat chewing presented with multiple traumas following a gunshot wound. The injuries included an open fracture of the left femur, a right posterior thigh skin defect, and a lower abdominal wall wound. Surgical reconstruction was performed using a type I keystone island perforator flap (KIPF) to cover both the thigh and abdominal defects. The patient showed satisfactory wound healing, with progressive scar maturation observed over time (Figure 5).

Patient Six

**Figure 6:**
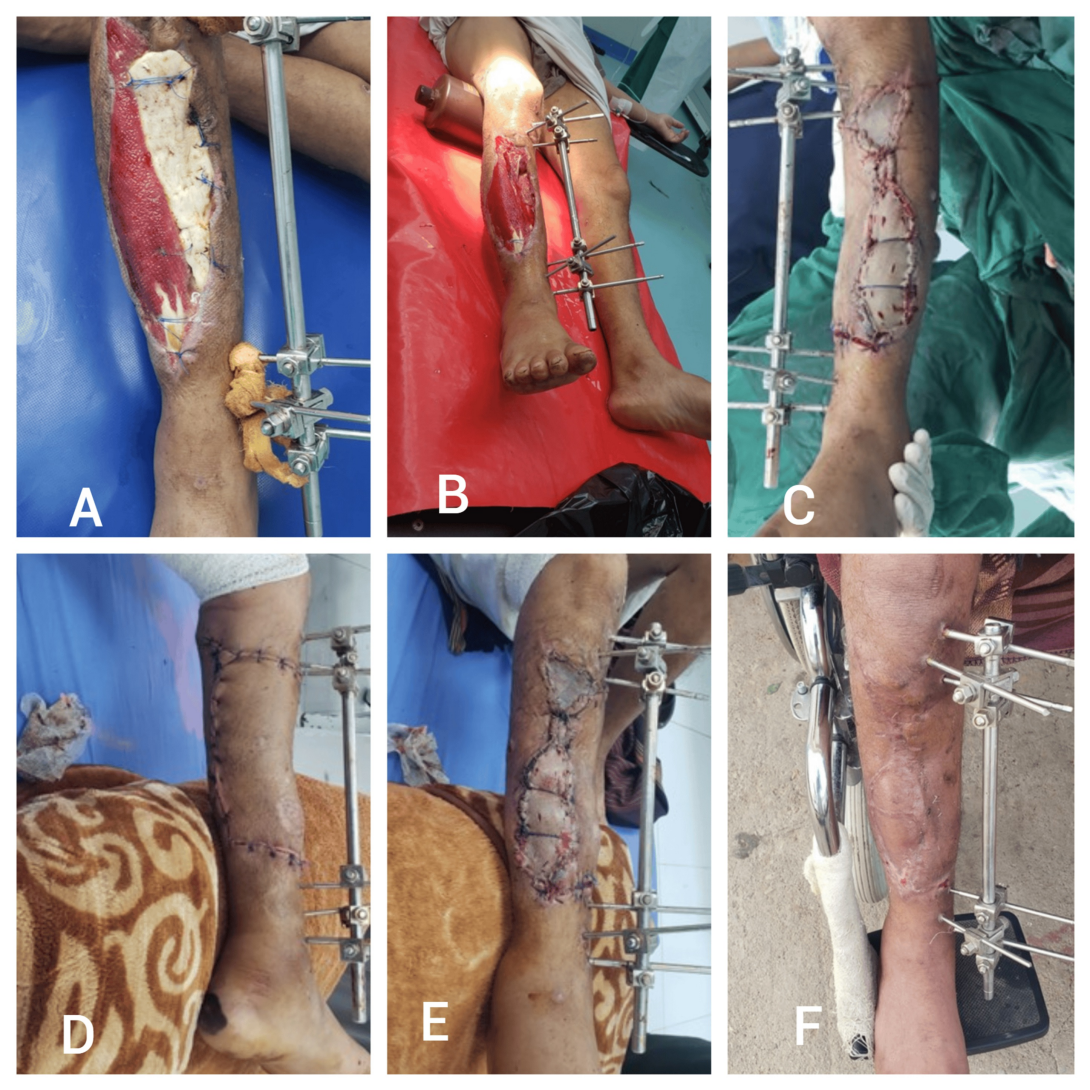
Patient six - a 41-year-old male patient sustained a gunshot injury that resulted in a proximal right tibial open fracture and a large skin defect. A) Preoperative image showing the large skin defect and exposed tibia following the gunshot injury; B) Intraoperative view demonstrating the extent of the defect and the exposed bone with the external fixator in place; C) Immediate postoperative view after the type 2A keystone island perforator flap (KIPF) was applied to cover the defect; D) Early postoperative image showing the initial healing of the flap; E) Further postoperative view indicating some areas of wound healing and minor complications being managed; F) Five-month follow-up showing successful flap integration and ongoing healing, with the external fixator still in place.

A 41-year-old male patient sustained a gunshot injury that resulted in a proximal right tibial open fracture and a large skin defect. Surgical intervention involved harvesting a type 2A keystone island perforator flap (KIPF) from the medial aspect of the proximal leg, with the flap extended posteriorly to enhance the vascularity of the proximal sural nerve. Postoperative follow-up at five months revealed successful flap integration and ongoing healing, with the external fixator remaining in place (Figure 6).

Patient Seven

**Figure 7:**
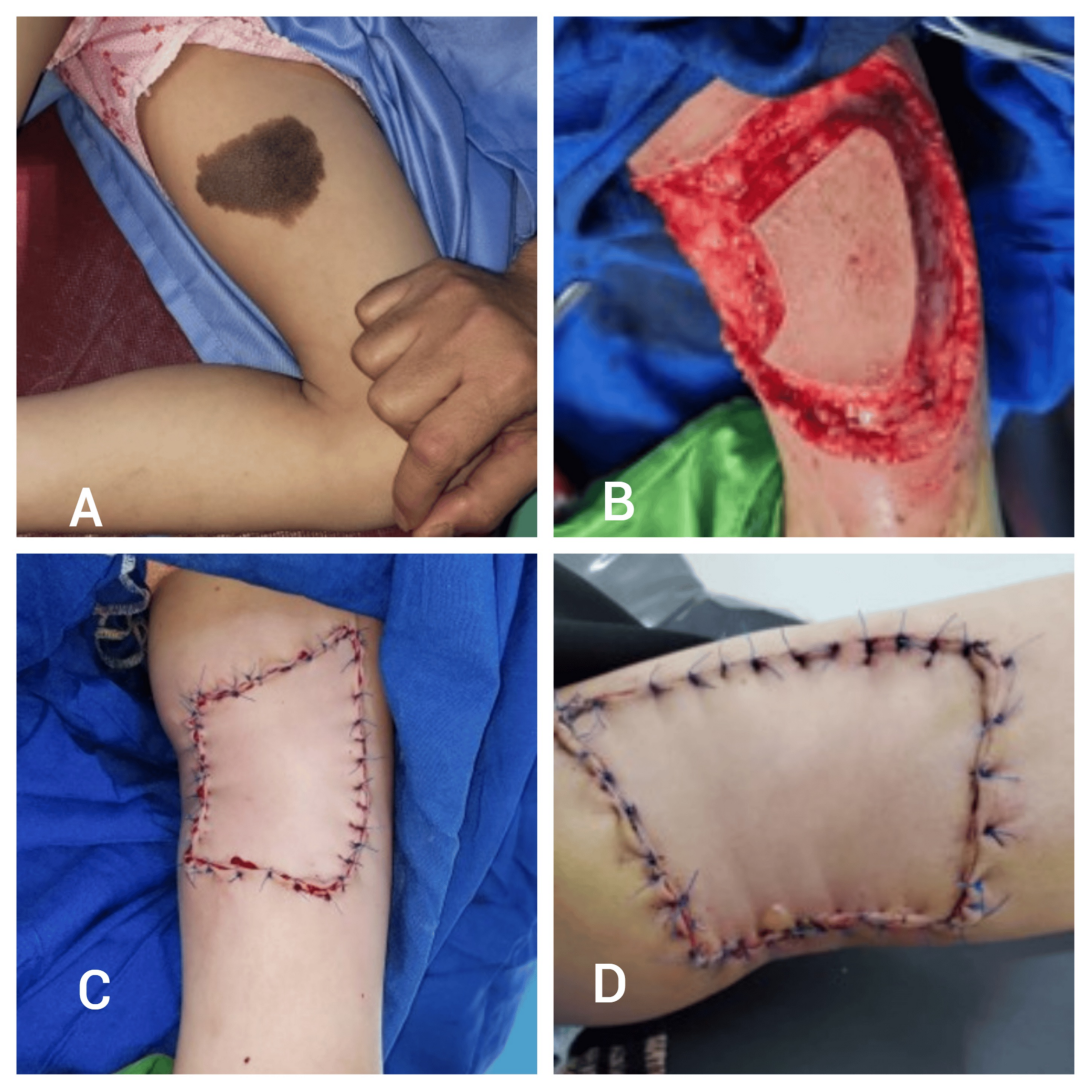
Patient seven - a 12-year-old girl presented with a congenital melanocytic nevus on the medial aspect of the left thigh A) Preoperative image showing the congenital melanocytic nevus on the medial aspect of the left thigh; B) Intraoperative view following the elliptical excision of the lesion, with the defect prepared for flap closure; C) Immediate postoperative view after the type 1 keystone island perforator flap (KIPF) was applied to close the defect; D) Early postoperative image showing satisfactory wound closure and healing progress.

A 12-year-old girl presented with a congenital melanocytic nevus on the medial aspect of the left thigh. Surgical intervention included the elliptical excision of the lesion, followed by the elevation of a type 1 keystone island perforator flap (KIPF) from the anterior side of the thigh to close the defect. The patient experienced favorable healing outcomes, with satisfactory wound closure observed by the fourth postoperative day (Figure 7).

Patient Eight

**Figure 8:**
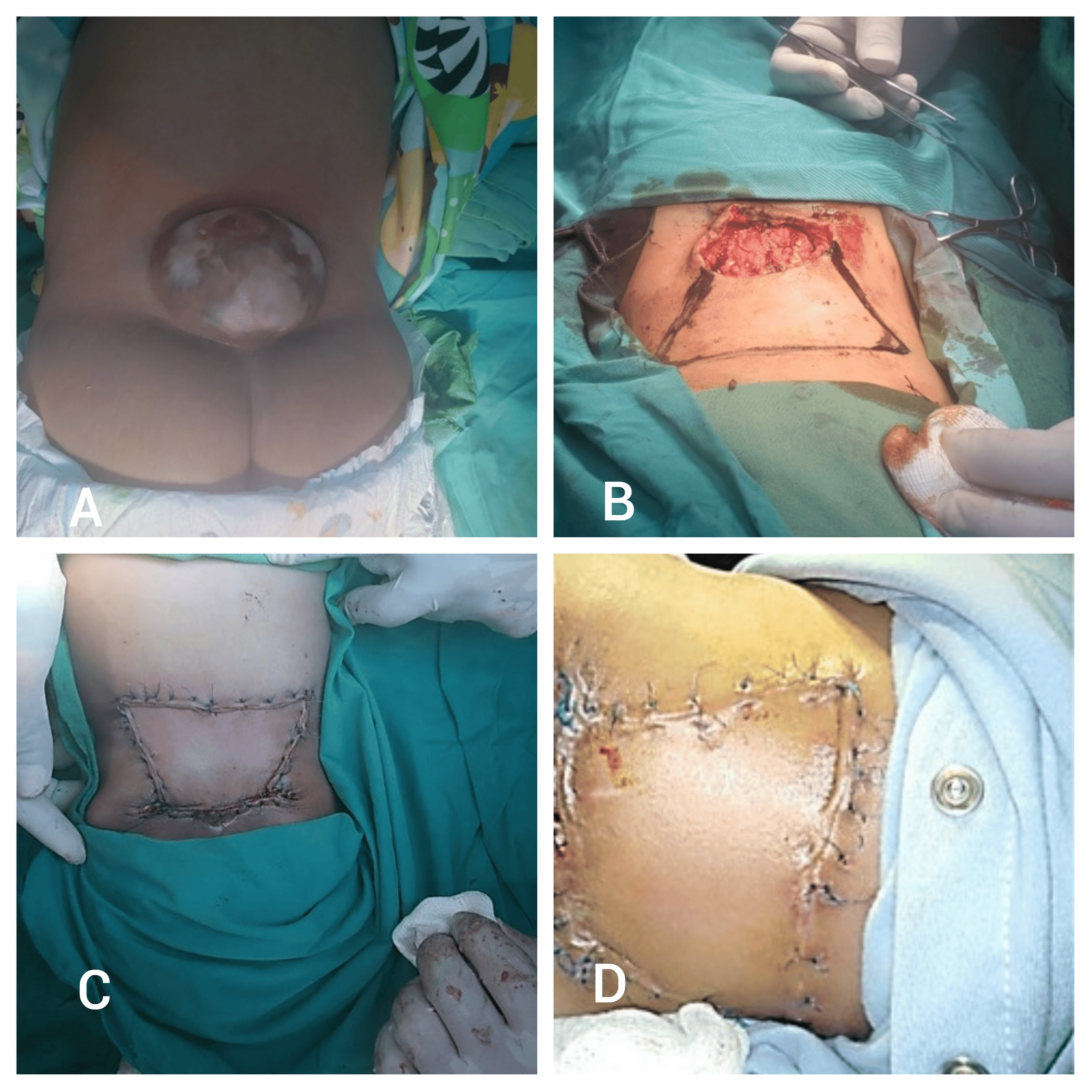
Patient eight - an 11-month-old female patient A) Preoperative image showing the myelomeningocele before surgical repair; B) Intraoperative view after neurosurgical repair, showing the defect prepared for the type 2A keystone island perforator flap (KIPF) closure; C) Immediate postoperative view following the flap closure, demonstrating successful defect coverage; D) Postoperative follow-up image at two weeks, indicating positive healing progress.

An 11-month-old female patient was diagnosed with myelomeningocele, which was surgically repaired by a neurosurgeon. The resulting defect was covered using a type 2A keystone island perforator flap (KIPF) designed along the lumbar nerve axis. The flap was advanced to close the defect, and the patient demonstrated positive healing progress at the two-week postoperative follow-up (Figure 8).

## Discussion

Our study revealed a high flap survival rate of 97% and a high wound healing rate of 91.4% within a median of 16 days. These outcomes are consistent with those reported by Khouri et al. (2011) [1], who reported a 97% reconstructive success rate for KIPF in patients with large trunk and extremity defects. Similarly, Huang et al. (2017) [15] highlighted the high success rates and low technical complexity of KIPF for lower extremity reconstructions.

The versatility of KIPF has been well documented across a range of clinical scenarios, including combat-related lower limb injuries [16], chronic wounds complicated by osteomyelitis [17], and head and neck reconstructions [2]. In our study, KIPF was successfully applied to various anatomical locations, primarily the legs (57.14%) and feet (11.43%).

Our postoperative outcomes, including high flap survival rates and minimal complications, align closely with the literature. For example, Weinberg et al. (2022) [18] reported a 98% overall success rate in a systematic review of KIPF applications, reinforcing the robustness of our findings. However, our study's complication rate of 26%, including flap dehiscence (11.43%) and wound infections, was higher than the 9.6% reported by Huang et al. (2017) [15]. This discrepancy might be attributed to the challenging clinical environment in Yemen, where advanced postoperative care may be limited.

In our study, scar quality assessments via the Patient Scar Assessment Scale (PSAS) and Observer Scar Assessment Scale (OSAS) revealed minimal differences from those of normal skin and satisfactory esthetic outcomes. These results are corroborated by Virág et al. (2022) [6], who reported that KIPFs provide effective wound closure with minimal donor site morbidity and high patient satisfaction. The reliability of the PSAS and OSAS has been validated by studies such as Bianchi et al. (2010) [19] and van der Wal et al. (2011) [20], emphasizing the reliability of these tools in evaluating scar quality.

Comparative studies have shown that KIPF offers several advantages over traditional free flaps, including shorter operation times, lower costs, and shorter hospital stays [21]. These benefits are particularly relevant in resource-limited settings. Modifications to the KIPF design, such as the bipedicled KPIF for chronic wound coverage [22] and the boat-shaped flap to reduce tension [23], have improved outcomes in complex wounds, including those resulting from large oncologic resections and irradiation [9].

Our study revealed that the majority of patients were young males (mean age 29 years), which is consistent with the findings of other studies focused on trauma-related reconstructions. The KIPF technique has been particularly beneficial for elderly patients, offering quick recovery times and low complication rates [3]. Factors affecting outcomes include comorbidities such as coronary artery disease, which is associated with increased rates of wound-healing complications and surgical site infections [2]. Despite the less favorable viscoelastic properties in patients with colored skin, the KIPF technique has been successfully applied, demonstrating its adaptability and effectiveness [24]. Additionally, Yoon et al. (2017) [25] reported promising results in treating pretibial defects in patients with comorbidities, further highlighting the versatility of the technique.

In our study, 80% of the patients did not have comorbidities, which may have contributed to the high flap survival rate and overall positive outcomes. The presence of comorbidities, such as diabetes mellitus and lower limb paralysis, noted in 5.7% of the participants, did not significantly impact the overall success of the KIPF procedures, which aligns with the findings of Yoon et al. (2017) [25] and Lanni et al. (2017) [2].

The KIPF has demonstrated promising long-term outcomes across various reconstructive procedures, reinforcing its reliability and effectiveness. In nasal defect reconstruction, KIPFs respect esthetic subunits, providing satisfactory outcomes at the 6-month follow-up [26]. Similarly, Formentin et al. (2019) [27] reported reliable results with no major complications in large myelomeningocele closures via KIPF. The technique has also proven effective in the management of melanoma, yielding pain-free and aesthetically pleasing results [28].

The efficacy of KIPFs is attributed to their unique design, which allows for tension redistribution and improved blood supply [29]. The UQ flap has further demonstrated favorable results for leg wound closure with minimal complications [5]. Behan (2008) [29] emphasized the reliability of fascially based flaps and the clinical principles underlying island flap efficacy, thereby reinforcing the long-term success of these techniques.

Studies have consistently shown high success rates for KIPFs, ranging from 95% to 98% [18]. Compared with skin grafts, flaps offer superior aesthetic outcomes, better donor-recipient color matching, and fewer contour defects [30]. Patients report high levels of satisfaction with both functional and cosmetic outcomes, a sentiment echoed by Yoon et al. (2017) [25], who documented favorable patient-reported outcomes across multiple anatomical locations.

In Yemen's resource-constrained setting, where there is no advanced center for microsurgical reconstruction and expertise in free flaps is limited, the KIPF technique has emerged as a promising local flap method in plastic surgery, offering better outcomes than skin grafts or secondary healing. By evaluating the success rate, aesthetic outcomes, influential factors, and complications, our research aimed to demonstrate the technique's increasing utility and safety, providing a viable solution to overcome the challenges of limited resources.

The positive esthetic outcomes in our study underscore the potential of keystone perforator island flaps for not only reliable wound closure but also satisfactory cosmetic outcomes. The flap design, which allows for local tissue rearrangement, minimizes the conspicuousness of the scar and contributes to excellent POSAS scores. The use of both observer- and patient-reported assessments in our study provides a comprehensive understanding of scar outcomes. The alignment of the observer and patient scores suggested a high level of agreement between the perceptions of the clinicians who assessed the scars and the patients who lived with the scars. This agreement strengthens the validity of the findings and underscores the importance of incorporating patient perspectives when assessing outcomes.

Limitations of the study

We should acknowledge several limitations, even though our prospective multicenter study offers valuable insights into the application of KPIF in a resource-constrained setting. The relatively small sample size of 35 patients may limit the precision of outcome estimates and the generalizability of the findings. Additionally, the absence of a control or comparison group restricts our ability to draw definitive conclusions regarding the relative effectiveness and advantages of KPIF compared to other reconstructive options.

The study's six-month follow-up period, although sufficient for assessing early to intermediate outcomes, may not capture long-term durability and potential late complications. Future studies with extended follow-up periods would be necessary to provide a more comprehensive evaluation of outcome stability over time. Furthermore, the study utilized the POSAS scale to assess aesthetic outcomes, but a more robust assessment that incorporates functional outcomes would enhance the comprehensiveness of the findings.

This study's unique context - a setting with limited access to advanced reconstructive techniques and infrastructure - differentiates it from similar research conducted in regions with more extensive resources. The generalizability of our findings to settings with more advanced surgical capabilities or different patient populations is uncertain. Nevertheless, our study highlights the practicality and utility of the KPIF technique under resource-limited conditions, where options like free flaps are not feasible. We warrant further research, ideally involving larger sample sizes, comparison groups, longer follow-up periods, and broader outcome measures, to fully elucidate the potential of KPIF across diverse settings and patient demographics.

## Conclusions

This study demonstrated the usefulness of KPIF for reconstructing various defects in a resource-constrained setting, highlighting its reliability, versatility, and ability to achieve excellent esthetic outcomes. These findings support the expanding utility and safety of KPIF for managing complex defects where microsurgical expertise is limited. Larger, longer-term studies with comparison groups are needed to fully establish the relative effectiveness and cost-effectiveness of KPIF.
